# Impact of Stroke Therapy Academic Industry Roundtable (STAIR) Guidelines on Peri-Anesthesia Care for Rat Models of Stroke: A Meta-Analysis Comparing the Years 2005 and 2015

**DOI:** 10.1371/journal.pone.0170243

**Published:** 2017-01-25

**Authors:** Aurelie Thomas, Johann Detilleux, Paul Flecknell, Charlotte Sandersen

**Affiliations:** 1 University of Liège, Faculty of Veterinary Medicine, Liege, Belgium; 2 University of Newcastle, Comparative Biology Centre, Newcastle, United Kingdom; Universita degli Studi di Napoli Federico II, ITALY

## Abstract

Numerous studies using rats in stroke models have failed to translate into successful clinical trials in humans. The Stroke Therapy Academic Industry Roundtable (STAIR) has produced guidelines on the rodent stroke model for preclinical trials in order to promote the successful translation of animal to human studies. These guidelines also underline the importance of anaesthetic and monitoring techniques. The aim of this literature review is to document whether anaesthesia protocols (i.e., choice of agents, mode of ventilation, physiological support and monitoring) have been amended since the publication of the STAIR guidelines in 2009. A number of articles describing the use of a stroke model in adult rats from the years 2005 and 2015 were randomly selected from the PubMed database and analysed for the following parameters: country where the study was performed, strain of rats used, technique of stroke induction, anaesthetic agent for induction and maintenance, mode of intubation and ventilation, monitoring techniques, control of body temperature, vascular accesses, and administration of intravenous fluids and analgesics. For each parameter (stroke, induction, maintenance, monitoring), exact chi-square tests were used to determine whether or not proportions were significantly different across year and p values were corrected for multiple comparisons. An exact p-test was used for each parameter to compare the frequency distribution of each value followed by a Bonferroni test. The level of significant set at < 0.05. Results show that there were very few differences in the anaesthetic and monitoring techniques used between 2005 and 2015. In 2015, significantly more studies were performed in China and significantly fewer studies used isoflurane and nitrous oxide. The most striking finding is that the vast majority of all the studies from both 2005 and 2015 did not report the use of ventilation; measurement of blood gases, end-tidal carbon dioxide concentration, or blood pressure; or administration of intravenous fluids or analgesics. The review of articles published in 2015 showed that the STAIR guidelines appear to have had no effect on the anaesthetic and monitoring techniques in rats undergoing experimental stroke induction, despite the publication of said guidelines in 2009.

## Introduction

According to the World Health Organisation, stroke remains the second most common cause of death in high-income countries [1]. One in four cases of stroke is fatal within a year in the UK, and the quality of life of stroke survivors is likely to be significantly impaired as over half of them are left with a disability [2]. Preclinical research aims to improve diagnosis and treatment of stroke patients, as reflected by the publication of hundreds of research papers every year. The principle underlying experimental stroke studies is relatively straightforward. Firstly, a focal cerebral ischemia is inflicted on rodents [3,4,5]. Secondly, some treatment is administered, and thirdly the infarct sizes are compared within treatment groups. There are nonetheless a near-infinite number of methodological variants, and little consensus regarding the ideal methodology to be used in experiments of this kind. Often studies involving animal models of acute cerebral ischemia fail to translate into human stroke treatments. Despite around 600 treatments having been reported as effective in preclinical studies [6], clinically proven treatment options are scarce, which suggests that further refinements may be required for rodent models to produce data relevant to human medicine.

The Stroke Therapy Academic Industry Roundtable (STAIR) has produced numerous recommendations [7,8,9,10,11,12] highlighting important issues in the experimental modelling of ischemic stroke. One document in particular, the STAIR guidelines on the rodent stroke model for preclinical stroke trials, published in 2009 [13], sought to promote the translation of animal studies to successful human stroke trials.

Briefly, the STAIR guidelines include recommendations related to various aspect of stroke models such as study design, therapeutic drug dose, choice of animal model, outcome measures, anaesthesia protocol and physiological monitoring.

The specific impacts of anaesthesia, respiratory depression, drugs and other factors such as core temperature are well documented in rodent models of stroke [14,15,16,17,18,19,20] and partly echoed in the STAIR guidelines [13]. The principal aim of this literature review is to document whether anaesthesia protocols (i.e., choice of agents, mode of ventilation, physiological support and monitoring) have differed since the publication of the STAIR guidelines in 2009 [13]. Further to the guidelines, we recorded the country where the study has been performed, the strain of rat that has been used in the study and the use of intra- or postoperative analgesia. Our working hypothesis was that anaesthetic management was not significantly different in peer-reviewed studies published in 2015 compared to 2005.

## Material and Methods

### Search strategy

The search was performed in December 2015 on MEDLINE (www.pubmed.com) using a previously published methodology [21]. Indexed and non-indexed papers were retrieved for two standardised search criteria: “stroke” and “rat”. All relevant entry terms for each search criterion were collected in the Medical Subject Heading database and are listed in Table 1. Search results for each search criteria were combined using the Boolean operator “AND”. A time filter was applied to the search results: only papers from 2005 (time filter: 01/01/2005-31/12/2005) and 2015 (01/01/2015-31/23/2015) were selected.

**Table 1:** List of Medical Subject Heading terms used for the search in MEDLINE.

**SSC**	Relevant MeSH terms
**Rat**	Rat; Rattus; Rattus norvegicus; Rats, Norway; Rats, laboratory; Laboratory Rat; Laboratory Rats; Rat, laboratory
**Stroke**	Apoplexy; CVA (Cerebrovascular Accident); CVAs (Cerebrovascular Accident), Cerebrovascular Accident; Cerebrovascular Accidents; Cerebrovascular Apoplexy; Apoplexy, Cerebrovascular; Cerebrovascular Stroke; Cerebrovascular Strokes; Stroke, Cerebrovascular; Strokes, Cerebrovascular; Vascular Accident, Brain; Brain Vascular Accident; Brain Vascular Accidents; Vascular Accidents, Brain; Cerebral Stroke; Cerebral Strokes; Stroke, Cerebral; Strokes, Cerebral; Stroke, Acute; Acute Stroke; Acute Strokes; Strokes, Acute; Cerebrovascular Accident, Acute; Acute Cerebrovascular Accident; Acute Cerebrovascular Accidents; Cerebrovascular Accidents, Acute

SSC: Standardized search criteria; MeSH: Medical Subject Headings.

### Eligibility and selection of the papers

Both sets of search results were screened for relevance and eligibility. All references corresponding to studies written in languages other than English were excluded. The remaining references were each attributed a number and random selection proceeded until 100 articles for each year had been chosen [random.org] according to the following criteria:

1. The full text of the manuscript was available online via the library of the University of Liège.
2. The reference corresponds to an original study. More specifically, literature reviews, books and book chapters, conference proceedings, reports, and guidelines were excluded.
3. Only studies reporting the use of adult or geriatric rats were included. Studies using neonatal rats, rats younger than eight weeks or weighing less than 220g were excluded.
4. The rats were used in an *in vivo* model of stroke.
5. The stroke model required surgery, and the rats were expected to regain consciousness after surgery.

Only references fulfilling all five criteria were retained for further analysis.

### Data extraction

Two authors (AT and CS) independently screened both sets of included references then reached consensus for each variable. The information on variables, as detailed in Table 2, was sought from each manuscript. Where the information was not reported in the manuscript, the corresponding data was recorded as ‘not reported' (NR).

**Table 2:** Factors extracted from the manuscripts included in the review.

	Factor	Final Categories or Unit	Type of Data
**Rat**	Strain	Name of strain	Category
**Stroke Model**	Type of stroke model	MCAO (alone or combined with other such as CCAO or 4VO)	Category
		Thrombosis	
		Cortical devascularisation	
		Other	
**Anaesthetic agents**	Name of the molecule(s) used to induce and maintain the anaesthetized state	Inhalants	Category
		Halothane	
		Isoflurane	
		Injectables	
		Chloral Hydrate	
		Barbiturates	
		Urethane combination	
		Ketamine combination	
		Other	
**Respiration and ventilation**	Nature of per-operative inspired gas	Room air or medical air	Category
		Medical air	
		O_2_	
		O_2_ enriched mixtures	
		O_2_/N_2_O	
		O_2_ enriched air	
	Mode of respiration	Spontaneous	Category, Binomial
		Mechanically Controlled or Assisted	
	Tracheal intubation	Yes/No	Category, Binomial
**Per-Anaesthesia Monitoring**	Temperature	Core temperature monitoring: Yes/No	Category, Binomial
		Heat pad: Yes/No	
	Heart Rate	Yes/No	Category, Binomial
	Blood Pressure	Invasive Blood Pressure (IBP): Yes/No	Category, Binomial
		Non-Invasive Blood Pressure (NIBP): Yes/No	
	Haemoglobin Saturation in Oxygen (S_p_O_2_)	Yes/No	Category, Binomial
	Blood parameters	P_a_CO_2_: Yes/No	Category, Binomial
		P_a_O_2_: Yes/No	
		pH: Yes/No	
		Glucose: Yes/No	
		Blood sampling (generic, no parameters mentioned): Yes/No	
**Per-Anaesthesia Fluid support**	Injectable fluids	Fluids administered: Yes/No	Category, Binomial
	Type of fluids administered	Name of Fluid	Category
	Volume administered	Volume in ml	Continuous
	Route of administration	IV: Yes/No	Category, Binomial
		SC: Yes/No	
		IP: Yes/No	
**Analgesia**	Drugs used to alleviate pain are mentioned	Yes/No	Category, Binomial
	Type of analgesic used	NSAIDs: Yes/No	Category, Binomial
		Paracetamol: Yes/No	
		Opioid: Yes/No	
		Local anaesthetic: Yes/No	
**Recovery**	Recovery from anaesthesia in heated environment	Yes/No	Category, Binomial

When a factor is not mentioned in the manuscript it was recorded as “NR” (not reported). *Whenever possible, a dose given in mg or ml per rat was converted to mg.kg^-1^. MCAO: Middle Cerebral Artery Occlusion; CCAO: Common Carotid Artery Occlusion; 4VO: Four Vessel Occlusion; F_i_: Inspired fraction; O_2_: oxygen; N_2_O: Nitrous Oxide; IBP: Invasive Blood Pressure; NIBP: Non-Invasive Blood Pressure; S_p_O_2_: heamoglobin saturation in oxygen; IV: Intravenous; IP: Intraperitoneal; SC: Subcutaneous; IM: Intramuscular; NSAID; Non-Steroidal Anti-Inflammatory drug.

### Statistical analysis

For each parameter (stroke, induction, maintenance, monitoring), exact chi-square tests were used to determine whether or not proportions were significantly different across year and p values were corrected for multiple comparisons. An exact p-test was used for each parameter to compare the frequency distribution of each value followed by a Bonferroni test. All the tests were performed using SAS 9.1 software with the level of significant set at p < 0.05.

## Results

**Fig 1:**
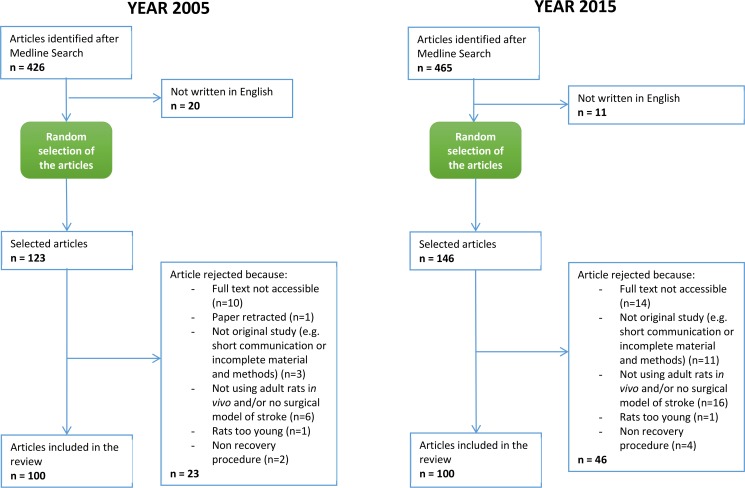
Flow of database search, screening, eligibility, selection and inclusion of original studies using a surgical rat model of stroke. Studies from 01/01/2005 to 31/12/2005 (A) and from 01/01/2015 to 31/12/2015 (B). See text for original search criteria.

The initial search delivered 426 hits for 2005 and 465 hits for 2015. Both sets of search results were screened for relevance and eligibility. All references corresponding to studies written in languages other than English were excluded (n = 20 for 2005, n = 11 for 2015). Remaining references were continually randomly selected and screened for inclusion/exclusion criteria until 200 publications (n = 100 for 2005, n = 100 for 2015) had been retained for further analysis. The number of papers rejected for various reasons is given in detail in Fig 1.

### Model

Focal brain ischemia produced by various means of MCAO was the most commonly used model. Significantly less MCAO was produced by techniques including thrombosis in 2015 (n = 0) than in 2005 (n = 17) (p = 0.0001).

### Anaesthetic agents

The STAIR guidelines offer the following guidance regarding the choice of anaesthetic agent:

“*When designing a preclinical study for neuroprotection*, *the protection provided by anesthetics should be taken into account*. *When neurotransmitters or neuroplasticity are the main foci of a study*, *anesthetics such as urethane*, *which do not disturb the action of neurotransmitters should be used*.*”* [13].

Anaesthetic agents used for induction and maintenance are listed in Table 3. In 2005, 79 studies did not report the induction agent, compared to 73 in 2015. Maintenance of anaesthesia (or the name of the chosen molecule) was not reported in 8 and 10 of the 100 reports analysed in 2005 and 2015, respectively. Halothane as a maintenance agent was significantly less often used in 2015.

**Table 3:** Agents for induction and maintenance of anaesthesia in rats used in stroke model studies in 2005 and 2015.

	2005	2015
**Induction Agent**		
Halothane	10	2
Isoflurane	10	20
Chloral Hydrate	1	0
Sevoflurane	0	1
Enflurane	0	1
Ketamine + Xylazine	0	2
Ether	0	1
Not reported	79	73
Total	100	100
**Maintenance Agent**		
Fentanyl-Fluanisone + Midazolam	1	0
Alpha Chloralose + Urethane	1	1
Barbiturates	7	6
Chloral Hydrate	20	27
Ether	1	1
Halothane	32	2*
Isoflurane	21	35
Ketamin +Xylazin	7	10
Methohexital sodium	1	1
Tribromoethanol	1	1
Enflurane	0	3
Sevoflurane	0	1
Tiletamin + Zolazepam	0	1
Tiletamin + Zolazepam + Xylazin	0	1
Not reported	8	10
Total	100	100

* significantly different from 2005.

### Intubation and ventilation

The STAIR guidelines offer the following guidance regarding the choice of anaesthetic agent:

“The importance of using mechanical ventilation should be determined by the anticipated impact of the surgical/anaestheic procedure on respiratory function. The potential confounding effects from respiratory functional deficits can be minimized by the use of mechanical ventilation. Unnecessary use of mechanical ventilation should be avoided when a particular MCAO model is not likely to cause respiratory problems.

*Ventilation may be needed when the operation lasts long (>1 hour) and when the ischemia affects brain stem function*. *A mixture of 30%*:*70% (O2*:*N2 or N2O) may be used for preclinical stroke trials combined with individualised adjustment of ventilator parameters”* [13].

The majority of the studies did not report the mode of ventilation (70 in 2005, 85 in 2015) or the use of oxygen supplementation (55 in 2005, 72 in 2015). In 2005, 17 studies reported spontaneous breathing, compared to 9 in 2015. Mechanical ventilation was reported in 13 studies in 2005 and 6 in 2015. There was a significant difference for the use of O_2_/N_2_O mixture, which was used in 36 studies in 2005 and 14 in 2015 (p = 0.0266). Some studies reported the use of O_2_-enriched air (6 in 2005, 3 in 2015), others 100% O_2_ (2 in 2005, 7 in 2015) or O_2_ and some, room air (1 in 2005, 4 in 2015).

### Anaesthesia monitoring and recovery

The STAIR guidelines offer the following guidance regarding blood sampling, blood pressure and core temperature monitoring:

*“Blood sampling is necessary for periodic measurement of arterial blood gas and frequency of measurement should be selected with reference to animal size*. *[…] Monitoring blood pressure during experiments is needed because blood pressure fluctuation affects stroke outcomes*. *Blood pressure can be monitored by non-invasive and invasive methods*. *Use non-invasive methods for experiments that cause minimal blood pressure fluctuation and require a neurological evaluation*. *Use invasive methods for experiments that require constant blood pressure monitoring*. *[…] Controlling animal body temperature in a normal range is necessary for eliminating the protective effect of hypothermia and potential harmful effect of hyperthermia*” [13].

The majority of all studies did not report if and how anaesthesia was monitored (71% in 2005, 80% in 2015). Nineteen studies in 2005 reported the use of arterial blood gas analysis and invasive blood pressure monitoring. This was significantly more commonly used than in 2015, where it was only reported in four studies (p = 0.0208). Control of body temperature was reported in 64 studies in 2005, and 65 in 2015.

Almost no data were found on the use of venous (86 in 2005, 97 in 2015) or arterial (74 in 2005, 87 in 2015) accesses; the administration of intravenous fluids (99 in 2005, 99 in 2015); or the control of temperature during recovery (90 in 2005, 92 in 2015). There was no significant difference in any of these parameters between 2005 and 2015.

### Country

All publications reported the country where the study had been performed. Studies had been performed in 26 different countries all over the world. In 2005, most studies were performed in the USA (n = 30). In 2015, significantly more studies had been conducted in China (n = 46) when compared to 2005 (n = 11) (p < 0.0001). Details of the number of studies performed per country in 2005 and 2015 are given in Table 4.

**Table 4:** Number of studies performed in a specific country.

Country	2005	2015
Argentina	0	1
Australia	1	1
Canada	5	1
China	11	46*
Denmark	0	1
Finland	2	2
France	2	3
Germany	11	3
Hungary	1	0
India	0	1
Iran	0	1
Ireland	0	1
Italy	1	3
Japan	17	4
Korea	6	7
Malaysia	0	1
Mexico	1	0
Poland	1	0
Slovakia	0	1
Spain	1	1
Sweden	5	2
Taiwan	3	4
Turkey	0	2
UK	2	1
Uruguay	0	1
USA	30	12

UK = United Kingdom, USA = United States of America.

* significantly different from 2005.

### Strain

Most of the studies reported the use of Sprague-Dawley rats for their research. This rat strain was used in 48 studies in 2005 and 72 studies in 2015. Wistar rats were the second most commonly used strain, with 34 studies in 2005 and 20 in 2015. There was no significant difference in the proportion of these two used strains across the two sample years. Other rat strains were Spontaneous Hypertensive Rats (7 in 2005, 3 in 2015), Long-Evans (7 in 2005, 2 in 2015), Wistar-Kyoto (3 in 2005, 1 in 2015), and Lister-Hooded (1 in 2005, 0 in 2015). Two papers from 2015 did not report the strain of rats used.

### Analgesia

The extreme majority of the studies did not report using analgesics (n = 96 in 2005, n = 94 in 2015). When described, various analgesic regimens were used: opioids (4 in 2005, 4 in 2015), non-steroidal anti-inflammatory drugs (1 in 2005, 0 in 2015), local anaesthetics (1 in 2005, 2 in 2015), local anaesthetics and opioids (0 in 2005, 1 in 2015), and local anaesthetics and paracetamol (0 in 2005, 1 in 2015).

## Discussion

Overall this review shows that anaesthetic techniques and their reporting for experimental stroke studies in rats have not changed significantly over the last ten years, despite the release of relevant guidelines in 2009 [13].

### Anaesthetic drug

Regarding anaesthesia protocols, the STAIR guidelines explain the neuroprotective role of most anaesthetics, their effect on neurotransmitters and receptors, and their potential effect on hyperglycemia. All these factors need to be taken into account when choosing the anaesthetic protocol for a study, and the protocol chosen can contribute to the bias of the results. The only difference in the choice of anaesthetic drug noticed between 2005 and 2015 is that halothane was less commonly used in 2015. This might be explained by the higher availability of isoflurane and isoflurane-specific equipment (i.e., vaporiser) rather than being a guideline-influenced choice of the researcher. Although not univocal, isoflurane and halothane have one-third less neuro-protective properties [22]. Some studies testing isoflurane in stroke models showed delay or improvement of the lesions [23,24,25] other studies suggest no collateral effect of isoflurane exposure during experimental stroke procedures [26]. However, available data suggests that lower doses of isoflurane in mild to moderate stroke do possess neuroprotective effects.

Similarly, results from different studies indicate conflicting results regarding the potential effect of halothane on ischemic brain injury. Some studies report that halothane anesthesia improved neurological outcomes during focal and global ischemia models [23,26,27,28]; while others failed to show an exacerbation of ischemic damage and neurological outcome [14,24,29].

The second most common drug for maintenance of anaesthesia was chloral hydrate, injected intraperitoneally (20% of the studies in 2005, and 27% in 2015). Intraperitoneal administration of chloral hydrate produces only light anaesthesia and may cause adynamic ileus, peritonitis, and gastric ulcers in rats. It is not recommended as sole anaesthetic agent and should be used in conjunction with barbiturates, opioids, alpha-2 agonists, or phenothiazine tranquillizers [30]. None of the papers reviewed in our study discussed the choice of drugs or mentioned anaesthesia as a limiting factor of the study.

The selection of the anaesthetic agent may also have an impact on cerebral blood flow (CBF), a crucial parameter during the development of the ischemic lesion. The amplitude of CBF increase depends on both the nature of the agent (halothane having a more severe effect than all other inhalants) and the dose administered [31]. Injectable agents, with the exception of ketamine, tend to decrease CBF [32]. As for the neuroprotective effect, none of the investigated studies discussed the possible consequences of the selected drug on the CBF and subsequent ischemic lesion. CBF is further determined by auto regulatory mechanisms and related to the animal's blood pressure, but only 14 and 21% of the studies (2015 and 2005, respectively) reported the use of blood pressure monitoring. Regardless of the use of such monitoring, none of the studies reported strategies to keep blood pressure in the desired range. As most studies do not mention using an intravenous access line or the administration of intravenous fluids, the pertinence of solely using of blood pressure monitoring remains questionable. However, blood pressure can be considered as a surrogate for anaesthetic depth; low blood pressure may invite the researcher to lower the concentration of inspired anaesthetic agents, when feasible [13].

### Ventilation/CO2 monitoring

Regardless of all the molecular modalities, ventilation is depressed during the anaesthetic phase in humans as well as animals. This is partly explained by the loss of respiratory muscles tone, changes in alveolar gas exchange [33,34], and by the blunting of the CO_2-_triggered ventilatory response [35,36].). In consequence, respiratory rhythm and tidal volume decrease and CO_2_ accumulates in the blood. As initially described by Grubb and collaborators [37], there is a significant linear relationship between P_a_CO_2_ and CBF. As long as the mean arterial pressure remains within physiological range, each mmHg increase in the P_a_CO_2_ causes a 1.8ml/100g/min change in the CBF. While originally demonstrated in non-human primates [37], this relationship exists across species. As a result, the anaesthetised state should be expected to influence both the size of the cerebral ischemia and its potential reperfusion, regardless of the experimental treatment being investigated. Tracheal intubation, controlled ventilation, and monitoring of arterial blood gases for rodent models of stroke are possible [14,38,39]. P_a_CO_2_ should be maintained within normal limits (35–45 mmHg), and CBF as close to physiological norms as possible [14]. However, our review suggests that only 6 and 13% of peer-reviewed stroke studies (2015 and 2005, respectively) used mechanical ventilation as part of the surgical phase of the protocol. Such findings conflict with previously published findings confirming the impact of hypercapnia on the size of cerebral injury [40], and the need for mechanical ventilation to control physiological variables [14], and reduce peri-operative mortality [20] in rat models of stroke. Numerous other scientific publications document the impact of P_a_O_2_, blood and CSF pH on CBF [41,42,43,44]. These CBF sensitivities are potential sources of noise when collecting data, suggesting the need for a wider sample size per treatment group than necessary under controlled anaesthetic conditions.

Reported monitoring techniques also included saturation of haemoglobin with oxygen and invasive and non-invasive blood pressure monitoring. The combination of invasive blood pressure monitoring and arterial blood gases being used was less common in 2015 than 2005. However, arterial blood gas analysis was more commonly measured in other combinations, which may suggest, that overall monitoring techniques have not changed over the years. Only 29 studies in 2005 and 20 studies in 2015 report any form of monitoring used.

### Temperature

Controlling body temperature within physiological range is crucial to avoiding the neuroprotective effect of hypothermia. Hypothermia reduces brain metabolism [45], while hyperthermia increases the metabolic rate of the brain and therefore the ischemic outcome [46]. In general, methods to control body temperature rely on measurement of rectal temperature, assuming a correlation between brain and rectal temperatures. However, several studies have shown considerable differences between rectal and brain temperatures [47]. Roughly, two thirds of all studies reported the use of body temperature monitoring or control during stroke induction, while the rest of the studies did not mention whether any form of temperature control was used or not. The STAIR guidelines underline the importance of body temperature control, not only during stroke induction but also during the recovery period. The most popular method is a warm chamber with a controlled temperature between 28–32°C. More sophisticated telemetric feedback systems also exist [48]. Only 8 (2005) and 7 (2015) studies report their animals recovering in a temperature-controlled environment.

### Model

Numerous reviews [19,49,50,51,52] have sought to appraise the characteristics and assumptions made during the creation and use of rodent models of stroke. However, the vast majority of these reviews focus on the refinement of the animal model itself (rodents vs. rabbits vs. other species); the modalities of cerebral ischemia (total vs. partial; permanent or not; arterial occlusion vs. embolisation vs. chemical vasoconstriction); appropriate windows for target drug administration; or presence of other confounding factors such as inflammation and neural regeneration. Rats are one of the most commonly used animals for stroke models [50]) partly because of the similarities between cerebral vasculatures and physiology with humans [53], simple husbandry and ease of restraint.

Because the middle cerebral artery (MCA) and its branches are most commonly responsible for cases of primary strokes in humans [54], middle cerebral artery occlusion (MCAO) is the most commonly used model of stroke induced in rats. Occlusion of the MCA can, however, be obtained in several ways. Briefly, it can either be directly occluded distally (after sub-temporal craniectomy) or cranially (occlusion of a carotid artery and thread of an occlusive filament from the chosen carotid to the MCA) [50,51]. Other indirect models are also in use, such as thrombosis, embolism, or the use of endothelin-I as a potent vasoconstrictive agent [50].

### Method/study limitations

The present review has some limitations, notably concerning the methodology. Although we tried to follow the search strategy developed by [Leenars and collaborators [21], there might have been areas open for improvement. Leenars and collaborators [21] say: *“to be* ***systematic***, *explicit and transparent*, *the scientist should always report*: *(1) all databases and other sources searched; (2) the dates of the last search for each database and the period searched; (3) full search strategies (including all search terms) for each database; and (4) any language or publication status restrictions used*.*”*. The present review only exploited one single database (PubMed, indexed and non-indexed papers) and only 100 randomly selected papers were analysed were analysed for each year. However, our aim was to identify methodological differences between two given years (2005 and 2015) and not to perform a complete meta-analysis of all rat stroke model papers published in the decade.

### Non-reporting

While the vast majority of papers provide statements on ethical review, the rat strain and the model used, most fail to report subsequently on anaesthetic and monitoring techniques, such as the use of arterial or venous accesses, ventilation, oxygen supplementation, or the administration of fluids or analgesics. While expected, this is a major finding. Therefore, it is possible that changes in anaesthesia protocols and techniques have occurred, but were not picked up by our study. For instance, 85% of the stroke studies included in our paper did not report on the mode of ventilation selected for their animal (i.e., spontaneous, assisted or controlled), and up to 96% failed to report whether analgesic agents were at all used peri-operatively. Such levels of non-reporting are very similar to a recent report by Carbone and Austin [55]. We did not attempt to assess whether the journal’s impact factor or commitment to publication guidelines [13,56] were associated with different levels of report since results from previous studies suggest that this is not the case [55,57,58], reinforcing the idea that guidelines may not be the golden grail to reduction of publication bias, better science and eventually more effective translational medicine [55,52,59,60,61].

### Non-implementation

Overall the results of the present review suggest that the publication of non-binding guidelines did not significantly influence the nature and quality of the anaesthetics and peri-operative monitoring used in studies of stroke models in rats. This disappointing result can be explained by a number of factors.

First, the advice provided by the guidelines may not be optimal. For instance, the STAIR guidelines stipulate that mechanical ventilation is “especially relevant during long operations (>1 hour) and when the ischemia affects brain stem function”, that “if the experiment is not likely to cause respiratory failure, intubation and mechanical ventilation may not be necessary”, and that “the intubation procedure itself and control of the mechanical ventilation process are technically demanding and may cause tissue damage even in experienced hands” [13]. Additionally, the guidelines fail to quote the studies linking hypercapnia and spontaneous ventilation with data variability, morbidity and mortality, despite publication long prior to the STAIR guidelines [41,42,43,44,40,14,20]. Despite these limitations, one might have expected that the STAIR recommendation to use mechanical ventilation would have triggered an increase in the use of mechanical ventilation and blood gas monitoring.

Second, clinical guidelines are not usually very effective in triggering a change in practice [62,63]. STAIR is an international working group composed of leading academic researchers, American government agencies and R&D representatives from industry (STAIR 2001). While STAIR is an acknowledged and respected working group, its influence may not have been sufficient per se to trigger the desired changes; in other words, guidelines do not implement themselves [64]. Assuming that the guidelines were communicated to the target audience of primary investigators involved in rat modelling of stroke, several barriers could have prevented their effective implementation. Four of the previously identified barriers are: the lack of familiarity with the guidelines; the lack of self-efficacy (i.e., the operator believes that s/he cannot perform the recommended action); the inability to overcome the inertia of previous practice (“we’ve always done it this way”); and the absence of external barriers to perform the recommendations (i.e., non-binding nature of the guidelines) [65]. Each of these barriers is relevant to the implementation of peri-anaesthesia refinements of laboratory animal care. Another important result from this study relates to the change in countries involved in rat stroke model studies. In particular, the number of studies published from China increased by 35% over the ten-year period. It seems very unlikely that the guidelines themselves are a causal factor associated with such relocation; multiple factors such as research funding availability, and flexibility of the legislative framework surrounding animal use are more likely to have contributed to this change. Other factors such as language and cultural barriers may have further diluted the influence of the STAIR guidelines in China compared to other parts of the world such as Europe.

Third, the evolution of anaesthetic protocols may have been impeded by the perceived technical difficulty/knowledge gap associated with some of the recommendations. The European Directive on the protection of animals used for scientific procedures (EU 2010/63) stipulates that all persons carrying out the procedures (users) have to be educated and trained before they can perform any task, and should be supervised in the performance of said task until they are proven competent (Article 23.2.c). In addition, all procedures must be disclosed in applications reviewed by the animal welfare and ethical review commission and approved by the competent authority. Such processes should, in theory, offer opportunities for the stakeholders to devise projects in accordance with available guidelines, identify potential knowledge/technical gaps, and help the people responsible for education and competence to point users towards bespoke additional training in advance of the launch of a project. The formal training mechanisms described above may, in some instances, fail to provide users with the required knowledge or skills for complete implementation of procedure-refinement guidelines. Performing scientific procedures involving animals can be very challenging for primary investigators, especially when the nature of the procedures to be undertaken are outside the researcher's field of expertise. Engineers can, for instance, find themselves needing to learn basic anaesthesia and surgical techniques to pursue the testing of a new type of CNS electrode in vivo. The person responsible for education and competence would be expected to point the engineer towards relevant additional training and support for the realisation of his/her procedures. Many would naturally look in the direction of the laboratory veterinarian for this.

Fourth, most would argue that scientists are not carrying sole accountability for the poor translatability of outputs from *in vivo* procedures and the disappointing impact of animal procedures on human medicine [66,67,68,58]. Funders, editors and reviewers all play a part in the genesis and dissemination of animal data [69]. Over 300 peer-review scientific publications have publicly endorsed the ARRIVE guidelines. Some of the major funding agencies (i.e. Wellcome Trust) make compliance with the ARRIVE guidelines a condition for funding [70]. In spite of this effort, two years after the publication of the ARRIVE guidelines there was no significant improvement in the quality of reporting of animal studies in top-tier journals (i.e. PLOS and Nature publishing groups) [71], suggesting that authors, but also referees, and maybe more surprisingly, editors, seems to ignore the guidelines they publicly embrace. Similarly the ARRIVE guidelines did not affect the reporting of anaesthetic and analgesic protocols for invasive animal studies [58], correlating with the fact that only 22% of peer-review critical care studies involving animals indicated using anaesthesia, analgesia and euthanasia [67].

Animal research is one of the most tightly regulated activities. Yet, in addition to the legislative framework, scientists need to comply with self-regulatory mechanisms such as biomedical journals’ policies on animal use. This supposes that policies must be implemented effect by editors and reviewers. While most people acknowledge that journals can, and should, help driving methodological changes [72] (Erb 2010), most journals restrict their involvement to recommending, without implementing reporting guidelines such as ARRIVE [71,70]. Recently, some landmark journals made additional efforts to counter this trend. In an attempt to increase the adherence to ethical use of animals and reporting, some journals have proposed a simplified, and easier to fill, version of the ARRIVE guidelines [73,74], others have developed their own compulsory and comprehensive animal ethics checklist [75], or organized discussion forums to address these issues [76]. It is worth noting that reviewers are usually chosen for their expertise in the scientific field of the study, and may lack the information, knowledge (or motivation) to assess whether a study is ethically sound beyond usual superficial statements. In consequence, this duty should revert to the editors, who would probably be expected to be competent in ethical assessment of the manuscripts [77,78].

Last, another explanation for the lack of implementation of animal research guidelines could sit within the discrepancy between the societal needs for improved human medicine (e.g. diagnostic tools, medical treatments) and the motivations of the scientists (e.g. high *h* and citation indexes). Numerous metric systems are used to measure the performance of individual scientists [79]. The vast majority of these indexes rely on the number of publications of study findings, the impact factor of the journal, and the number of citations of the published study. Although systematic ranking of scientific performance was initially developed to help to boost discoveries [80], most scientists now believe that metrics of performance are being used in hiring, promotion decisions, performance review and funding attribution. It could, therefore, be argued that the scientists' initial vocational motivation to solve scientific problems and help improving human health may be outweighed by the need to demonstrate performance through repeated publication of scientific findings in high impact factor journals.

We argue that, should the scientists’ primary incentive shift from the number and impact factor of their publications to the production of accurate fundamental knowledge or its contribution towards the resolution of a human medical condition, the likelihood of obtaining relevant reproducible and translatable data from *in vivo* scientific procedure would improve. We propose that scientific recognition could be attributed based on the quality of the study design, ability to reproduce the published findings and contribution towards a medical application rather than number of publication and the impact factor of the journal. If such incentive shift was implemented, primary investigators might also become more inclined to actively refine their procedures in an attempt to control potential confounding factors (e.g., anaesthesia, pain and distress) and maximise the signal-to-noise ratio within their data. Similarly, if scientists were judged based on the reproducibility of their findings, they may take better care in reporting every step of their intervention, including anaesthetic and analgesic protocols, as well as the fate of each animal allocated to the treatment groups. In other words, the authors argue that not only the shift of incentive described above would contribute towards improving the quality and relevance of animal research, it would also contribute to add value to the services offered by competent laboratory animal veterinarians and the numerous support guidelines already available to the scientific community.

## Conclusion

In conclusion, this study suggests that despite the publication of rodent-specific guidelines for models of stroke, anaesthetic modalities were similar in 2015 to those in 2005. Non-binding guidelines alone are unlikely to trigger practical and efficient changes in the way laboratory animals are anaesthetised. The intrinsic nature of the guidelines as well as the inability to implement suggested changes in research institutions may be causal factors. In particular, the practical expertise required for the application of research guidelines may not be readily available.
